# COVID-19-Related Fear and Anxiety: Spiritual-Religious Coping in Healthcare Workers in Portugal

**DOI:** 10.3390/ijerph18010220

**Published:** 2020-12-30

**Authors:** Filipe Prazeres, Lígia Passos, José Augusto Simões, Pedro Simões, Carlos Martins, Andreia Teixeira

**Affiliations:** 1 Faculty of Health Sciences, University of Beira Interior, 6200-506 Covilhã, Portugal; jars58@gmail.com (J.A.S.); pedro.agrm.simoes@gmail.com (P.S.); 2 Family Health Unit Beira Ria, 3830-596 Gafanha da Nazaré, Portugal; 3 Centre for Health Technology and Services Research (CINTESIS), University of Porto, 4200-450 Porto, Portugal; ligiamaria@ua.pt (L.P.); carlosmartins20@gmail.com (C.M.); andreiasofiat@med.up.pt (A.T.); 4 Department of Education and Psychology, University of Aveiro, 3810-193 Aveiro, Portugal; 5 Institute of Biomedical Sciences Abel Salazar, University of Porto, 4050-313 Porto, Portugal; 6 Family Health Unit Caminhos do Cértoma, 3050-428 Pampilhosa, Portugal; 7 Family Health Unit Pulsar, 3030-790 Coimbra, Portugal; 8 MEDCIDS—Department of Community Medicine, Information and Decision in Health, Faculty of Medicine, University of Porto, 4099-002 Porto, Portugal; 9 Polytechnic Institute of Viana do Castelo, 4900-347 Viana do Castelo, Portugal

**Keywords:** COVID-19, health personnel, anxiety, fear, coping skills, religion, spirituality

## Abstract

The COVID-19 pandemic has negatively affected the mental health of the general population, and for healthcare workers (HCWs) it has been no different. Religiosity and spirituality are known coping strategies for mental illnesses, especially in stressful times. This study aimed to describe the role of spiritual-religious coping regarding fear and anxiety in relation to COVID-19 in HCWs in Portugal. A cross-sectional quantitative online survey was performed. Socio-demographic and health data were collected as well as the Duke University Religion Index, Spirituality Scale, Fear of COVID-19 Scale, and Coronavirus Anxiety Scale. Two hundred and twenty-two HCWs participated in the study, 74.3% were female and 81.1% were physicians. The median age was 37 years (Q1, Q3: 31, 51.3). Religiosity was neither a significant factor for coronavirus-related anxiety nor it was for fear of COVID-19. Participants with higher levels in the hope/optimism dimension of the Spirituality Scale showed less coronavirus-related anxiety. Female HCWs, non-physicians, and the ones with a previous history of anxiety presented higher levels of fear and/or anxiety related to COVID-19. HCWs’ levels of distress should be identified and reduced, so their work is not impaired.

## 1. Introduction

Since the World Health Organization (WHO) declared the COVID-19 pandemic on 11 March 2020 [1], the world lives an unprecedented scenario, where one acute disease brought disruption in several aspects of the society, such as health (physical and mental), economy, social security, environment, among others [2]. More than 47 million people around the world have been infected with the SARS-CoV-2 virus and over 1477 million deaths have been reported from the disease [3]. In Portugal, 300,462 cases were registered, with 4577 deaths until 1 December 2020 [4].

It is recognized that the aforementioned pandemic has negatively affected the mental health of the general population [5]. For healthcare workers, it was not different. For example, in China, a cross-sectional study of 1257 hospital workers exposed to COVID-19, demonstrated that symptoms of depression, anxiety, insomnia, and distress were common, especially in front-line female nurses [6]. These results were further supported by very recent literature review [7]. This review also found that fear of uncertainty or becoming infected was one of the biggest challenges healthcare workers had to cope with [7]. Other factors such as the frequent growth of COVID-19 cases and deaths due to the disease, lack of resources (material and human), unspecific and ineffective treatments, in addition to an increase in work demands are known to contribute to healthcare workers’ exhaustion and mental health deterioration [8,9].

The adult population in Portugal is mostly Catholic (77%) and the importance of religion in Portuguese people’s lives is extensive, 44% are absolutely certain that God exists, 36% state that religion is very important in their lives, 36% attend religious services at least monthly, and 37% pray every day [10].

The terms religiosity and spirituality may be similar at first, but their definitions express different ideas. “The common ground that religion and spirituality share is a search for the sacred through the experience of subjective feelings, thoughts, and behaviors” [10]. Religiosity refers to a person’s behavior and attitudes in relation to a specific religion and its rules, values, and practices, and can be measured by the actions of attending religious temples, praying, and reading sacred scriptures [11,12]. Religiosity can be divided into two distinct types: intrinsic religiosity that refers to the affinity between personal beliefs/values and the religious ones; with having a strong bond with the embraced creed [13,14]; extrinsic religiosity refers to the use of religion to supply personal basic needs (e.g., social relations or personal comfort) and keeping the focus on oneself [13,15].

Spirituality can be defined as how individuals seek and manifest meaning and purpose in life; how they connect with the moment, themselves and others, with nature, and in relation to the sacred [16]. It can also be understood as an internal belief system, which brings vitality and meaning to life’s events [17]. Examples of spiritual support include “meditation, relaxation, listening to music, and guided imagery” [18].

Religious/spiritual people are considered to be healthier both physically and mentally [19,20]. Religiosity and spirituality are known coping strategies for both physical and mental illnesses [11,17,21,22], especially in stressful crises [23]. When religious beliefs, attitudes, or practices are used to reduce emotional stress caused by life events that are beyond personal control, there is spiritual-religious coping, which gives meaning to suffering, making it more bearable [24]. A positive religious coping has been associated with a reduction of depression and anxiety, as well as with increased psychological well-being [25], and patients with high intrinsic religiosity have more depression remissions [21]. In contrast, negative religious coping is linked to negative psychological adjustment to stress [23]. Spirituality is also associated with having a positive effect on adjusting to disability [26]. In a recent cross-sectional study in the Brazilian general population, a high spiritual-religious coping during the COVID-19 pandemic was associated with higher levels of hopefulness and lower levels of fear, worrying, and sadness [27].

Among healthcare workers, the role of spiritual-religious coping is less known. In the US, providers who attended religious services at least once per week had a lower risk of dying from despair, compared with those who had never attended [28].

Some authors consider that the COVID-19 pandemic may be an ideal scenario to trigger spiritual-religious coping in society [29]. In Portugal, there is still a lack of mental health research regarding religiosity and spirituality [30]. Therefore, the objective of this study was to describe the role of spiritual-religious coping regarding fear and anxiety in relation to COVID-19 in healthcare workers in Portugal.

## 2. Materials and Methods

### 2.1. Study Design

A cross-sectional quantitative survey, using an electronic questionnaire, was performed from July to August 2020 amongst healthcare professionals working in Portugal.

### 2.2. Setting and Participants

For inclusion in the study, participants had to be healthcare workers (physicians, dentists, nurses, pharmacists, psychologists, gerontologists, and medical secretaries) working in Portugal, gave their informed consent, and agreed to participate in the study. The survey was created in Google Forms platform and took approximately five minutes to complete. The research team used their own professional contacts to send the survey weblink to prospective participants via email (e.g., groups of religious healthcare professionals and professional societies) and to professional groups in social media networks. Subsequently, research participants also recruited other participants for the study—snowball sampling.

### 2.3. Measurements

Sociodemographic data, such as gender, age, marital status, with or without children, profession, and professional status were collected. Data on clinical aspects were also collected: Diagnosis of COVID-19, personal history of anxiety, and if the healthcare worker had direct contact with patients with or suspected of COVID-19.

Duke University Religion Index (DUREL): A 5-item instrument that measures the religious involvement divided into three subscales (dimensions of religiousness): organizational religious activity (ORA)—question 1; non-organizational religious activity (NORA)—question 2; and subjected or intrinsic religiosity (IR)—questions 3 to 5. Response options are on a 5-point or 6-point Likert scale, depending on the question(s), and the overall score ranges from 5 to 27 [31]. The utilization of the resulting overall index is not recommended in research, and so the three subscales should be used separately [14]. The internal consistency (Cronbach’s alpha) ranged from 0.78 to 0.91 [14]. In the Portuguese validated version alpha values ranged from 0.733 for the total scale, to 0.758 for the intrinsic religiosity subscale [32].

Spirituality Scale (SS): created in Portugal to assess spirituality in health contexts [33], comprising 5 items rated on a Likert 4-points scale. It is subdivided into two dimensions: “beliefs” (items 1 and 2) and “hope/optimism” (items 3 to 5); the score for each dimension is calculated by averaging the score of its items. A high score on each item indicates a high degree of agreement with the assessed dimension. The internal consistency for the global scale is 0.74, being 0.92 for the beliefs dimension and 0.69 for the hope/optimism dimension [33].

Fear of COVID-19 Scale (FCV-19S): developed and published by Ahorsu et al. in March 2020 [34], facing the needs imposed by the pandemic, this 7-item scale is a brief measure for investigating the fear of COVID-19. Each item must be answered on a Likert 5-points scale (from 1 = strongly disagree to 5 = strongly agreeing). By adding up each item score, the total index ranges from 7 to 35 points, and the higher the score, the greater the fear of COVID-19. In terms of reliability, the scale has an internal consistency of 0.82 [34].

Coronavirus Anxiety Scale (CAS): Developed and published by Lee in April 2020 [35]. This scale makes a quick mental health screening, to identify levels of anxiety triggered by the COVID-19 pandemic. It consists of five items that refer to usual anxiety symptoms and each item is answered with a five-point Likert-type scale (from 0 = not at all to 4 = nearly every day). It has excellent reliability, with a Cronbach’s alpha of 0.93 in the original Lee’s study [35]. The CAS is currently being translated into several languages and is already in use by a large number of health professionals and researchers worldwide [36].

To the best of the authors’ knowledge, at the time of the present study’s protocol design, no Portuguese translation was available for either FCV-19S or CAS. Consequently, both FCV-19S and CAS had to be translated from English to Portuguese. We used the translation methodology that our team usually follows: First, the English-language items of both instruments were initially translated by a panel of five general practitioners that were native Portuguese speakers well-versed with the English language. Afterwards, the translations were reconciled by the authors after discussion and till a consensus was achieved. The final version of both instruments was back-translated by a native English speaker. It was pilot tested in 12 health-related professionals and no alterations to the instruments were needed. In the present study, Cronbach’s alpha (internal consistency) for FCV-19S was 0.83 and for CAS was 0.86.

### 2.4. Statistical Analysis

Data analysis was performed using SPSS 26.0 version statistical software (IBM, Armonk, NY, USA). Categorical variables were described by absolute and relative frequencies, n (%) or (n; %). Continuous variables not normally distributed, or ordinal variables were described using the median and the interquartile interval, Mdn (Q1, Q3). The normality of continuous variables was verified by observation of the respective histograms.

The comparison of quantitative variables between physicians and other healthcare workers was made by the Mann–Whitney test, since the variables were ordinal variables or continuous variables not normally distributed. The association between quantitative variables was analyzed by the Spearman coefficient (r_s_).

Separated multiple linear regressions were performed for fear of COVID-19 Scale (FCV) and Coronavirus Anxiety Scale (CAS) as dependent variables. To determine which variables (gender, age, profession, with children, personal history of anxiety, direct contact with patients with or suspected of COVID-19 and the variables resulting from the DUREL and SS scales) must be included in each multiple models as independent variables, simple linear regressions were realized. All variables that correlated with the dependent variables at *p* ≤ 0.20 in a simple regression were included in the multiple linear regressions and only the significant variables were included in the final multiple models. The results of linear regressions were described using the coefficient values (β) and the respective p-value. The determination coefficient (r^2^) of the model was indicated to evaluate it. The assumptions of the linear regression models were assessed by observation of histograms to verify the normality of residuals and using plotting residuals versus the fitted predictive values for checking homoscedasticity. The assumptions of the linear regression models were assessed by observation of histograms to verify the normality of residuals; plotting residuals versus the fitted predictive values for checking homoscedasticity and values of tolerance and variance inflation factor (VIF) to detect problems of multicollinearity (if VIF > 10 or tolerance < 0.1). Values of *p* ≤ 0.05 were considered significant.

### 2.5. Sample Size

The minimum sample size (*n* = 196) was calculated for proportions and considering the most conservative scenario (a proportion of 50%), a population of 150,990 healthcare professionals in Portugal [37], a level of confidence of 95% and an error margin of 7%.

### 2.6. Ethics

The study was reviewed and approved by the Ethics Committee of the University of Beira Interior (CE-UBI-Pj-2020-052) and followed the Declaration of Helsinki ethical standards. Electronic consent was obtained from all participants. Responses were anonymous.

## 3. Results

Two hundred and twenty-two healthcare workers filled the online questionnaire completely and were included in the analysis. The sociodemographic and health data of the participants are summarized in Table 1; 180 (81.1%) were physicians and 42 (18.9%) other healthcare workers The majority of the participants were female (165; 74.3%); median age was 37 years (Q1, Q3: 31, 51.3), and 132 (59.5%) were married or had a civil partnership. More than half of the sample had direct contact with patients with or suspected of COVID-19, and 8 participants tested positive for COVID-19. Almost one-third of the sample (32.9%) had a personal history of anxiety.

Regarding Religiosity, the scores obtained by the Duke University Religion Index (DUREL) were: Organizational Religious Activity (ORA) median 4 (Q1, Q3: 2, 5) points; Non-Organizational Religious Activity (NORA) 4 (Q1, Q3: 1, 5) points; and Intrinsic Religiosity (IR) 12 (Q1, Q3: 9, 14) points, and there were no significant differences between physicians and other healthcare workers (*p* = 0.709, *p* = 0.633, *p* = 0.515, respectively; Table 2). Considering the Spirituality Scale, respondents scored above average, with a median Beliefs score of 3 (Q1, Q3: 2, 4) points and a median Hope/Optimism score of 2.83 (Q1, Q3: 2.33, 3.33) points, also without significant differences between physicians and other healthcare professionals (*p* = 0.509, *p* = 0.622, respectively; Table 2).

The median score of the Fear of COVID-19 Scale was 14 (Q1, Q3: 11; 17) points and non-physicians had a significantly higher median score than physicians (*p* = 0.001; Table 2).

Regarding the Coronavirus Anxiety Scale, the median score was 0 (Q1, Q3: 0, 2) points and non-physicians had a slight but significantly higher median score than physicians (1 vs. 0, *p* = 0.006; Table 2).

A Spearman’s correlation was run to determine the relationship between Religiosity and Spirituality and the values of Fear and Anxiety of COVID-19 (Table 3). There was a very weak, negative correlation between the subscale of Hope/Optimism and anxiety of COVID-19, which was statistically significant (r_s_ = −0.191; *p* = 0.004; Table 3). Thus, higher values of Hope/Optimism are associated with lower coronavirus anxiety in healthcare professionals.

As shown in Table 4, gender, profession, and personal history of anxiety were found to be significant factors for Fear of COVID-19 in multiple linear regression (r^2^ = 0.117). Women’s Fear of COVID-19 was higher by an average of 2.14 in comparison to men (*p* = 0.004). The healthcare professionals, who were not physicians, had levels of Fear of COVID-19 higher by an average of 2.66 in comparison to physicians (*p* = 0.001). Those who had a personal history of anxiety were significantly associated with increased levels of Fear of COVID-19 (β = 1.51, *p* = 0.024). There were no collinearity problems in the variables included in the final model (gender, tolerance: 0.95, VF: 1.06; profession, tolerance: 0.96, VF: 1.05 and personal history of anxiety, tolerance: 0.99, VF: 1.01). The histogram of standardized residuals showed that the data contained approximately normally distributed errors and the plot of standardized predicted values checked the homoscedasticity property.

It can be seen from the data in Table 5 that gender, personal history of anxiety, and Hope/Optimism were found to be significant factors for coronavirus-related anxiety in multiple linear regression (r^2^ = 0.105). Women’s coronavirus-related anxiety was higher by an average of 0.98 in comparison to men (*p* = 0.007). Respondents who had a personal history of anxiety were significantly associated with increased levels of coronavirus-related anxiety (β = 1.18, *p* < 0.001). Higher levels of Hope/Optimism were significantly associated with a reduction of coronavirus-related anxiety levels (β = −0.41, *p* < 0.043). There were no collinearity problems in the variables included in the final model (gender, tolerance: 0.99, VF: 1.01; personal history of anxiety, tolerance: 0.97, VF: 1.03 and hope/optimism, tolerance: 0.98, VIF: 1.02). However, the histogram of standardized residuals showed that the distribution is slightly skewed on the right and the plot of standardized predicted values indicated heteroscedasticity.

## 4. Discussion

The main purpose of the current study was to describe the role of spiritual-religious coping regarding fear and anxiety in relation to COVID-19 in healthcare workers in Portugal. This is, to the best of the authors’ knowledge, the first study to analyze such a topic in Portugal.

In the present sample of Portuguese healthcare employees, with similar levels of religiosity and spirituality between physicians and non-physicians, and contrary to expectations, it was observed that religiosity was neither a significant factor for coronavirus-related anxiety nor it was for fear of COVID-19. This finding is conflicting to previous studies that have suggested that religiosity offers cognitive and emotional tools to deal with uncertainty and to overcome adversity [29,38,39]; others have proposed that religiosity is positively related to mental health issues [28]. The inconsistency of the present results regarding previous studies may be due to the fact that since healthcare workers continued to perform their duties during the COVID-19 pandemic (and in most cases even had to work extra hours) [40,41]. They may have distanced themselves from religious-group-related activities and consequently the support obtained through their religious communities may have been reduced. The hypothesis of social distancing from religious activities was also pointed out in a recent study conducted in people who have been affected by the COVID-19 pandemic and in which no relationship was found between mental health and the different dimensions of religiosity measured by the Duke University Religion Index [42]; these results are consistent with the current study data. Another reason could be that the high personal and work-related burnout found in Portuguese healthcare workers during the COVID-19 pandemic [42] neutralized the positive effects that religion had on mental health in previous studies.

Nonetheless, spirituality was associated with a lower coronavirus-related anxiety. Participants with higher levels in the hope/optimism dimension of the Spirituality Scale showed less coronavirus-related anxiety. This finding could indicate that when healthcare workers have to cope with anxiety, the sense of hope, and the positive expectancies regarding the self, the others and the environment are tools they use to reduce anxiety levels [33,43]. The dimension beliefs that were mostly linked with the practice of religion, involved neither a significant factor for coronavirus-related anxiety, nor it was for fear of COVID-19 which is consistent with the results found for religiosity with the Duke University Religion Index. Although the Terror Management Theory proposes that religion helps people cope with death [44], COVID-19 pandemic might have influenced religious beliefs and thus increased anxiety. For example, people who have higher levels of religiosity may believe in God as a punisher and those less religious may believe that there is no God [45].

In the present study, being a female healthcare worker was a predictor of higher levels of both coronavirus-related anxiety and fear of COVID-19. This result is in agreement with recently published studies conducted in the general population of several countries [46,47,48], including Portugal [5]. In these studies, the female gender was found to be more vulnerable psychologically than males during the COVID-19 pandemic, with a higher prevalence of anxiety and/or fear.

Several reports have shown that healthcare workers’ fears during the COVID-19 pandemic included the fear of being infected and rapidly spreading the disease to others (especially to family members), and the fear of not diagnosing COVID-19 positive patients [6,49,50]. In this study, the healthcare professionals, who were not physicians, expressed higher levels of COVID-19 related fear. An implication of this is the possibility that if non-physicians are too overburdened by fear then their work may not be executed properly and because of that they should be identified and supported as soon as possible [51]. Despite that, not only the healthcare professionals with higher levels of distress should have formal support, but should also the ones with previous history of anxiety, since it may increase vulnerability to healthcare providers’ fear and anxiety during the current COVID-19 pandemic [51].

### Limitations

This paper has some limitations worth noting. First, the results gathered by the non-probability sampling technique may be somewhat limited since they may not generalize to all healthcare workers in Portugal. Second, for privacy reasons, it was not asked which was the religion of the respondent. This might be of interest since religious coping may be different among diverse religions [52]. Third, the instruments used to measure the outcomes of fear and anxiety related to COVID-19 were not previously validated in the Portuguese population. Although, this was expected since both were very recent tools, in the current study both instruments presented good internal consistency (Cronbach’s alpha >0.8). Fourth, the final model presented for the outcome anxiety related to COVID-19 did not fulfill all the assumptions of Linear Regression, thus, these data need to be interpreted with caution. However, this article is an exploratory approach, and in future studies, it will be necessary to select more variables (e.g., number of years of professional experience, academic qualifications, professional categories, working settings), especially because the model determination coefficient is low (r^2^ = 0.105). Last, self-report measures may not be the best way to investigate spirituality and religiosity, as they involve complex feelings and beliefs. Self-reports are subject to some biases and limitations, such as, social-desirability bias [53]. In future investigations, it might be interesting to explore this topic through other methods, as the expressive writing, method used to communicate intense emotions during catastrophic events [54,55].

## 5. Conclusions

The role of spiritual-religious coping regarding fear and anxiety in relation to COVID-19 in healthcare workers in Portugal is limited. Only individuals with higher levels of hope/optimism showed less coronavirus-related anxiety. No protective factors for fear of COVID-19 were found. Female healthcare workers, non-physicians, and the ones with a previous history of anxiety may experience higher levels of distress during the COVID-19 pandemic and therefore should be identified and supported as soon as possible so their work is not impaired and their quality of life improved. Also, the authors consider that the delivery of out-of-hours online religious and spiritual support for healthcare workers may be a viable solution to not only potentiate spiritual-religious coping during COVID-19 in this population but also to offer relational support that is primarily human.

## Figures and Tables

**Table 1:** Socio-demographic and health data (*n* = 222).

Characteristics	
Gender, *n* (%)	
Female	165 (74.3)
Male	57 (25.7)
Age (years), Mdn (Q_1_, Q_3_)	37 (31; 51.25)
Marital Status, *n* (%)	
Single	77 (34.7)
Married/Civil partnership	132 (59.5)
Widowed	13 (5.9)
Professional status, *n* (%)	
Employed	214 (96.4)
Unemployed	2 (0.9)
Retired	6 (2.7)
Profession, *n* (%)	
Physician	180 (81.1)
Other healthcare worker	42 (18.9)
With children, *n* (%)	
Yes	126 (56.8)
No	96 (43.2)
Diagnosis of COVID-19, *n* (%)	
Yes	8 (3.6)
No	214 (96.4)
Personal history of anxiety, *n* (%)	
Yes	73 (32.9)
No	149 (67.1)
Direct contact with patients with or suspected of COVID-19, *n* (%)	
Yes	147 (66.2)
No	75 (33.8)

**Table 2:** Comparison between the 2 groups (physicians vs. other healthcare workers).

Variables	Total (*n* = 222)	Physicians (*n* = 180)	Other Healthcare Workers (*n* = 42)	*p*-Value
Organizational Religious Activity (ORA), Mdn (Q_1_,Q_3_)	4 (2; 5)	4 (2; 5)	3 (2.75; 5)	0.709 ^a^
Non-Organizational Religious Activity (NORA), Mdn (Q_1_,Q_3_)	4 (1; 5)	4 (1; 5)	4 (1; 5)	0.633 ^a^
Intrinsic Religiosity (IR), Mdn (Q_1_,Q_3_)	12 (9; 14)	12 (9; 14)	13 (9; 14)	0.515 ^a^
Beliefs, Mdn (Q_1_,Q_3_)	3 (2; 4)	3 (2; 4)	3.5 (2.38; 4)	0.509 ^a^
Hope/Optimism, Mdn (Q_1_,Q_3_)	2.83 (2.33; 3.33)	3 (2.33; 3.33)	2.67 (2.33; 3.33)	0.622 ^a^
Fear of COVID-19 Scale, Mdn (Q_1_,Q_3_)	14 (11; 17)	13 (11; 17)	16.5 (13.75; 22)	0.001 ^a^
Coronavirus Anxiety Scale, Mdn (Q_1_,Q_3_)	0 (0; 2)	0 (0; 1)	1 (0; 3)	0.006 ^a^

a: Mann–Whitney test.

**Table 3:** Spearman correlation between outcomes (Fear of COVID-19 Scale and Coronavirus Anxiety Scale) and the subscales (ORA, NORA, IR, Beliefs and Hope/Optimism).

	Fear of COVID-19 Scale	Coronavirus Anxiety Scale
Organizational Religious Activity (ORA)	−0.039; *p* = 0.562	−0.071; *p* = 0.292
Non-Organizational Religious Activity (NORA)	0.023; *p* = 0.730	−0.020; *p* = 0.762
Intrinsic Religiosity (IR)	0.040; *p* = 0.555	0.001; *p* = 0.984
Beliefs	0.059; *p* = 0.379	−0.055; *p* = 0.411
Hope/Optimism	−0.075; *p* = 0.269	−0.191; *p* = 0.004

**Table 4:** Multivariable Model—Fear of COVID-19 Scale (FCV-19S).

	Initial Model (r^2^ = 0.124)		Final Model (r^2^ = 0.117)	
	FCV-19S	*p*-Value	FCV-19S	*p*-Value
Gender				
Male	Reference		Reference	
Female	2.11 (0.67; 3.55)	0.004	2.14 (0.70; 3.59)	0.004
Profession				
Physician	Reference		Reference	
Other healthcare worker	2.61 (1.01; 4.21)	0.002	2.66 (1.06; 4.26)	0.001
Personal history of anxiety				
No	Reference		Reference	
Yes	1.51 (0.19; 2.84)	0.025	1.51 (0.20; 2.82)	0.024
Intrinsic Religiosity	0.04 (−0.24; 0.32)	0.779	-	
Beliefs	0.26 (-0.80; 1.32)	0.628	-	

**Table 5:** Multivariable Model—Coronavirus Anxiety Scale (CAS).

	Initial Model (r^2^ = 0.119)		Final Model (r^2^ = 0.105)	
	CAS	*p*-Value	CAS	*p*-Value
Gender				
Male	Reference		Reference	
Female	0.83 (0.12; 1.54)	0.022	0.98 (0.27; 1.67)	0.007
Profession				
Physician	Reference			
Other healthcare worker	0.76 (-0.03; 1.55)	0.060		
Personal history of anxiety				
No	Reference		Reference	
Yes	1.15 (0.50; 1.81)	<0.001	1.18 (0.53; 1.84)	<0.001
Hope/Optimism	−0.40 (−0.80; −0.01)	0.045	−0.41 (−0.80; −0.01)	0.043

## Data Availability

The data presented in this study are available on request from the corresponding author. The data are not publicly available due to ethical re-strictions.
